# Development and validation of a coagulation-related genes prognostic model for hepatocellular carcinoma

**DOI:** 10.1186/s12859-023-05220-4

**Published:** 2023-03-09

**Authors:** Wan-Xia Yang, Hong-Wei Gao, Jia-Bo Cui, An-An Zhang, Fang-Fang Wang, Jian-Qin Xie, Ming-Hua Lu, Chong-Ge You

**Affiliations:** 1 Laboratory Medicine Center, Lanzhou University Second Hospital, Lanzhou, China; 2 Anesthesiology Department, Lanzhou University Second Hospital, Lanzhou, China

**Keywords:** Hepatocellular carcinoma, Coagulation-related gene, Prognosis, Risk score

## Abstract

**Background:**

Hepatocellular carcinoma (HCC) has a high incidence and mortality worldwide, which seriously threatens people's physical and mental health. Coagulation is closely related to the occurrence and development of HCC. Whether coagulation-related genes (CRGs) can be used as prognostic markers for HCC remains to be investigated.

**Methods:**

Firstly, we identified differentially expressed coagulation-related genes of HCC and control samples in the datasets GSE54236, GSE102079, TCGA-LIHC, and Genecards database. Then, univariate Cox regression analysis, LASSO regression analysis, and multivariate Cox regression analysis were used to determine the key CRGs and establish the coagulation-related risk score (CRRS) prognostic model in the TCGA-LIHC dataset. The predictive capability of the CRRS model was evaluated by Kaplan–Meier survival analysis and ROC analysis. External validation was performed in the ICGC-LIRI-JP dataset. Besides, combining risk score and age, gender, grade, and stage, a nomogram was constructed to quantify the survival probability. We further analyzed the correlation between risk score and functional enrichment, pathway, and tumor immune microenvironment.

**Results:**

We identified 5 key CRGs (FLVCR1, CENPE, LCAT, CYP2C9, and NQO1) and constructed the CRRS prognostic model. The overall survival (OS) of the high-risk group was shorter than that of the low-risk group. The AUC values for 1 -, 3 -, and 5-year OS in the TCGA dataset were 0.769, 0.691, and 0.674, respectively. The Cox analysis showed that CRRS was an independent prognostic factor for HCC. A nomogram established with risk score, age, gender, grade, and stage, has a better prognostic value for HCC patients. In the high-risk group, CD4^+^T cells memory resting, NK cells activated, and B cells naive were significantly lower. The expression levels of immune checkpoint genes in the high-risk group were generally higher than that in the low-risk group.

**Conclusions:**

The CRRS model has reliable predictive value for the prognosis of HCC patients.

**Supplementary Information:**

The online version contains supplementary material available at 10.1186/s12859-023-05220-4.

## Background

Global cancer statistics in 2020 indicates that there are approximately 906,000 new cases and 830,000 deaths of primary liver cancer worldwide [1]. Primary liver cancer is the sixth most common cancer and the third most common cause of cancer-related deaths worldwide, with hepatocellular carcinoma (HCC) accounting for 75–85% of primary liver cancers. The incidence and mortality of liver cancer continue to increase compared to the statistics of about 841,000 new cases and 782,000 deaths of primary liver cancer worldwide in 2018 [2]. Due to the insidious onset of HCC, most patients are diagnosed at an advanced stage of the disease, so the best treatment time is lost. The prognosis is extremely poor, causing a serious economic burden, and seriously threatening people's physical and mental health. In addition, the pathogenesis of HCC remains unclear. Therefore, identifying key molecules associated with HCC, understanding the molecular mechanisms of HCC, and finding therapeutic targets will contribute to the early diagnosis, treatment, and prognosis of HCC.

Tumor patients usually show a hypercoagulable state [3]. Studies have found that the risk of venous thromboembolism (VTE) in tumor patients is 9 times higher than in the general population [4], which seriously affects the survival rate of tumor patients. The incidence of portal vein thrombosis (PVT) caused by HCC is as high as 40% [5]. PVT is an indicator of the aggressiveness of HCC and can be used as an important predictor of the poor prognosis of HCC patients [6]. Studies [7] have found that the tissue factor (TF) of extrinsic coagulation pathway can promote HCC tumorigenesis. Von Willebrand factor antigen (vWF-Ag) can be used as a prognostic marker for postoperative outcomes of HCC patients, which is closely related to the incidence of postoperative complications and long-term outcomes [8]. Fibrin-like protein 1(FGL1) is a member of the fibrinogen-associated protein (FREP) family. The expression level of FGL1 is related to the progression and prognosis of HCC [9]. Therefore, we focused on the prognostic value of coagulation-related genes (CRGs). Furthermore, studies have demonstrated the coagulation cascade plays an important role in the tumor immune microenvironment (TME) [10].

TME is closely related to tumor generation, survival, and metastasis of HCC [11]. Tumor coagulome is a molecular effector network driven by cancer in which many genes and proteins jointly promote the balance between coagulation and fibrinolysis. Tumor coagulome and its regulation have become a hot topic in cancer research [12]. Therefore, the detection of coagulation function can provide a clinical basis for early diagnosis of HCC, which is of great significance for reducing the risk of postoperative death in HCC patients.

At present, few studies have explored the prognosis and immune status of HCC from the perspective of CRGs. In this study, we screened CRGs for HCC by multiple datasets and Genecards database. A coagulation-related risk score (CRRS) prognostic model for HCC was constructed based on CRGs in the TCGA dataset and validated for predictive ability in the ICGC dataset. Further, we analyzed the correlation between risk score and clinicopathological indicators. Then, combined with risk score and other prognostic clinical indicators, a nomogram model was constructed to quantify the survival probability. Finally, the relationship between risk score and functional enrichment, as well as TME was studied. This study provides a molecular basis for the complex mechanism of HCC and a potential target for the treatment of HCC.

## Materials and methods

### Data download and sample information collection

The expression profile data and corresponding platform annotation information of microarray datasets GSE54236 and GSE102079 were downloaded from the GEO database (https://www.ncbi.nlm.nih.gov/geo/). The GSE54236 dataset included 81 HCC tissues and 80 cirrhotic non-malignant tissues. A total of 152 HCC tissues and 105 control tissues were included in the GSE102079 dataset. We also downloaded clinical information data and RNA-seq data from The Cancer Genome Atlas (TCGA) database (https://portal.gdc.cancer.gov/) and International Cancer Genome Consortium (ICGC) database (https://dcc.icgc.org/). There were 374 HCC samples and 50 control samples in the TCGA-LIHC dataset, and 243 HCC samples and 202 control samples in the ICGC-LIRI-JP dataset. When analyzing the prognostic value of CRGs for HCC, some cases with incomplete clinical data were excluded. A total of 370 patients were included in the TCGA-LIHC dataset and 232 patients were included in the ICGC-LIRI-JP dataset (Additional file 1: Table S1). In addition, 4332 CRGs were retrieved from the Genecards database (https://www.genecards.org/) using the keyword "coagulation related genes". We validated the expression of key CRGs in LIHC tissues and normal tissues in the UALCAN database (http://ualcan.path.uab.edu/analysis.html).

### Obtaining the differentially expressed coagulation-related genes

We firstly converted the probes into gene symbols through the corresponding platform annotation information. The GSE54236, GSE102079, and TCGA-LIHC datasets were normalized by using "limma" package. We finally identified the differentially expressed genes (DEGs) with the cut-off conditions of |log_2_FC|> 1 and adjusted *P* < 0.05. The volcano plot of DEGs was drawn by using the “ggplot2” package. Additionally, a Venn diagram of differentially expressed coagulation-related genes (DECRGs) was drawn for visualization.

### Construction of the coagulation-related genes prognostic model

We firstly used R software package "survival" for univariate Cox regression analysis to identify the genes that were significantly associated with survival by calculating the relationship between DECRGs and overall survival (OS) in the TCGA-LIHC dataset. Then, the selected genes were further screened by LASSO regression with the R package "glmnet". The variation in regression coefficients of the prognostic genes was identified by selecting the optimal and minimal criteria of the penalization parameter. Subsequently, multivariate Cox regression analysis helped to determine the key CRGs and establish the CRRS prognostic model. Akaike information criterion (AIC) was used to measure the accuracy and brevity of the model. The model with the lowest AIC value was considered to be the most simple and effective model with the least information loss when predicting the result. The concordance index (C-index) was also calculated as a measure of accuracy in predicting survival outcomes.

### Tumor tissue collection and qPCR detection

A total of 18 human HCC and 16 adjacent tissues were collected in the Department of General Surgery, Lanzhou University Second Hospital. All experiments involving human tissues complied with the principles of the Declaration of Helsinki and have been approved by the Medical Ethics Committee of Lanzhou University Second Hospital. The total RNA was extracted by using TRNzol Reagent, and was reverse-transcribed with FastKing gDNA Dispelling RT SuperMix (TIANGEN, Beijing, China). All qPCR reactions were conducted with RotorGene 6000 PCR system (Qiagen) and performed with SsoFast EvaGreen Supermix (Bio-Rad). The relative expression of the gene was calculated by the 2^−ΔΔCt^ method. The primers used were shown in Additional file 2: Table S2.

### Development and validation of the coagulation-related risk score model

A total of 370 patients were divided into high-risk and low-risk groups based on median risk score (0.936) in the TCGA-LIHC dataset. The accuracy of the CRRS model was evaluated by Kaplan–Meier survival curve and ROC curve. We used the "survminer" package to draw Kaplan–Meier survival curve to analyze the relationship between risk score and OS. The package "survival" and "timeROC" were used to draw the ROC curve. The AUC value of the ROC curve was calculated to evaluate the performance of the prognostic model. In addition, the "pheatmap" package was used to draw risk curve, survival status plot, and heatmap of model genes in high-risk and low-risk groups. The ICGC-LIRI-JP dataset was employed for external validation of the prediction effect of the model.

### Construction and validation of the clinical prognostic model

Univariate and multivariate Cox regression analyses were conducted on the TCGA and ICGC datasets to evaluate whether the CRRS can be used as an independent prognostic factor. To quantify the survival probability of patients, a nomogram integrating the risk score and age, gender, grade, and stage was constructed by the "rms" package. Simultaneously, a decision curve analysis (DCA) was performed with "ggDCA" package to determine the clinical application value of the risk score model by calculating the net benefits. Further, the calibration curve was drawn to evaluate the predictive accuracy of the nomogram.

### Enrichment analysis

To understand the functions of DEGs between the high- risk and low-risk groups, we used the "org.Hs.eg.db" and "clusterProfiler" packages to conduct the Gene Ontology (GO) enrichment analysis and Gene Set Enrichment Analysis (GSEA). GO enrichment analysis includes biological process (BP), cellular component (CC) and molecular function (MF). Adjusted *P* < 0.05 was considered as statistically significant.

### Risk score and TME

The CIBERSORT database (http://cibersort.stanford.edu) provides a computational method for estimate immune cell composition from gene expression profiles of large numbers of samples. Combined with CIBERSORT, the R software was used to transform gene expression profile data into the proportion data of immune cells corresponding to samples, run with 1000 permutations. The R software “fmsb” package was used to draw radar map of immune cell. The ssGSEA algorithm with the “gsva” package was used to determine the differences in immune function between the high-risk and low-risk groups. We further compared the correlation between the risk score and the expression levels of immune checkpoint genes. The Wilcoxon test was employed to contrast the successive variates between the two groups.

### Statistical analysis

The Wilcoxon test was employed to contrast the successive variates between the two groups. The Kruskal–Wallis test was used for multiple comparisons. The chi-squared test was used for the comparison of categorical variable data between the two groups. Hazard ratios and 95% Confidence Interval were calculated using univariate and multivariate Cox analyses. All statistical* P* values were two-sided. *P < 0.05 was considered to be statistically significant. Statistical analyses were performed with the R 4.1.0 software and the GraphPad Prism 8.0.1 software.

## Results

### Identification of DECRGs

**Fig. 1:**
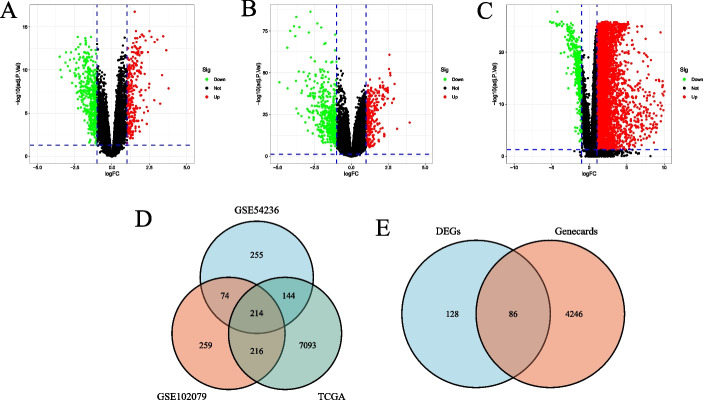
Identification of DECRGs. **A** The volcano plot of DEGs in the GSE54236 dataset. **B** The volcano plot of DEGs in the GSE102079 dataset. **C** The volcano plot of DEGs in the TCGA-LIHC dataset. **D** The Venn diagram of share DEGs among GSE54236, GSE102079, and TCGA-LIHC datasets. **E** The Venn diagram of DECRGs

To obtain DEGs of HCC and control group, we analyzed the GSE54236, GSE102079 and TCGA-LIHC datasets with a Bayesian test. The screening criterion was | log_2_FC |> 1 and adjusted *P* < 0.05. In the GSE54236 dataset, 687 DEGs (including 255 up-regulated genes and 432 down-regulated genes) were found (Fig. 1A). 763 DEGs were identified to be differentially expressed in the GSE102079 dataset, among which 284 were up-regulated and 479 were down-regulated (Fig. 1B). In the TCGA-LIHC dataset (Fig. 1C), 7667 DEGs were identified (including 7273 up-regulated genes and 394 down-regulated genes). There were 214 overlapping DEGs in the three datasets (Fig. 1D). A total of 4332 CRGs were obtained in Genecards database. After intersecting the DEGs with CRGs, we obtained 86 DECRGs (Fig. 1E).

### Construction of the prognostic model based on key CRGs

**Fig. 2:**
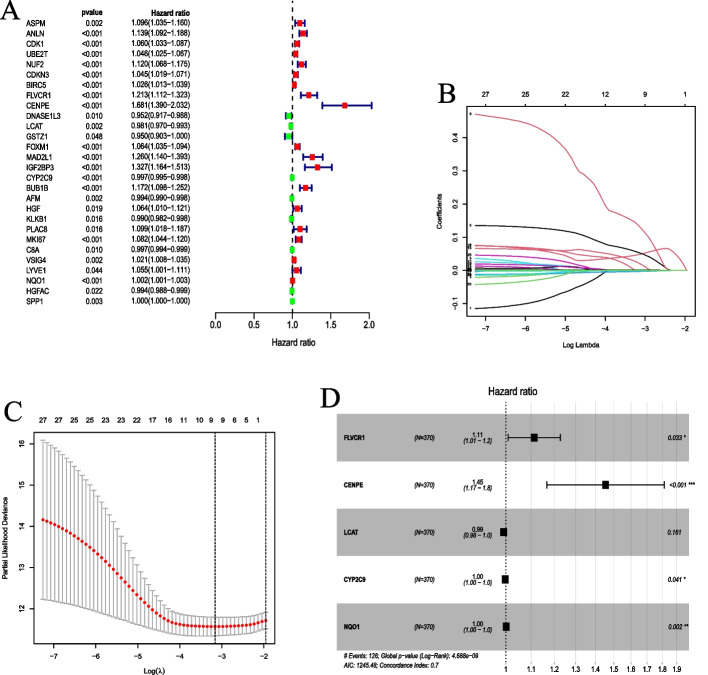
Identification of a CRRS prognostic model for HCC. **A** The forest plot of prognostic CRGs identified by univariate Cox analysis. **B** Cross validation for tuning parameter selection in the LASSO regression analysis. **C** The 9 key CRGs were selected by the LASSO regression analysis

To identify the key CRGs with prognosis significance, we conducted univariate Cox regression analysis and screened out 28 genes significantly related to the survival of HCC with *P* < 0.05 in the TCGA dataset, of which 19 were risk factors and 9 were protective factors (Fig. 2A). Further, 9 genes were obtained by LASSO regression analysis with the minimal penalization parameter. (Fig. 2B–C). Finally, 5 key CRGs were screened by multivariate Cox analysis and a CRRS prognostic model was constructed with AIC value is 1245.48 and C-index is 0.7 (Fig. 2D). The 5 key CRGs were as follows: FLVCR1, CENPE, LCAT, CYP2C9, NQO1. The prognostic model showed that FLVCR1, CENPE and NQO1 were risk factors, while LCAT and CYP2C9 were protective factors. The coefficient of CRGs was presented in Table1. The CRRS prognostic model for calculating HCC risk score was as follows: risk score = (0.1065 * FLVCR1) + (0.3748 * CENPE) + (− 0.0082 * LCAT) + (− 0.0019 * CYP2C9) + (0.0019 * NQO1).

**Table 1:** The coefficient of the 5 genes

Gene symbol	Gene description	Coefficient
FLVCR1	Feline leukemia virus subgroup C cellular receptor 1	0.1065
CENPE	Centromere protein E	0.3748
LCAT	lecithin-cholesterol acyltransferase	− 0.0082
CYP2C9	Cytochrome P450, family 2, subfamily C, polypeptide 9	− 0.0019
NQO1	NAD(P)H dehydrogenase, quinone 1	0.0019

**Fig. 3:**
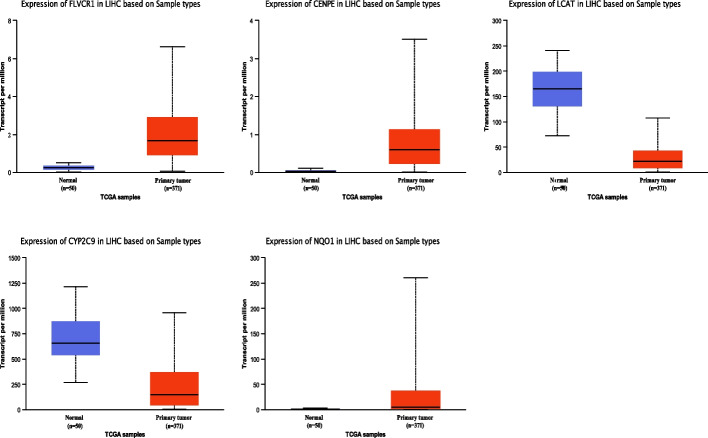
The expression levels of key CRGs in HCC and normal tissues

**Fig. 4:**
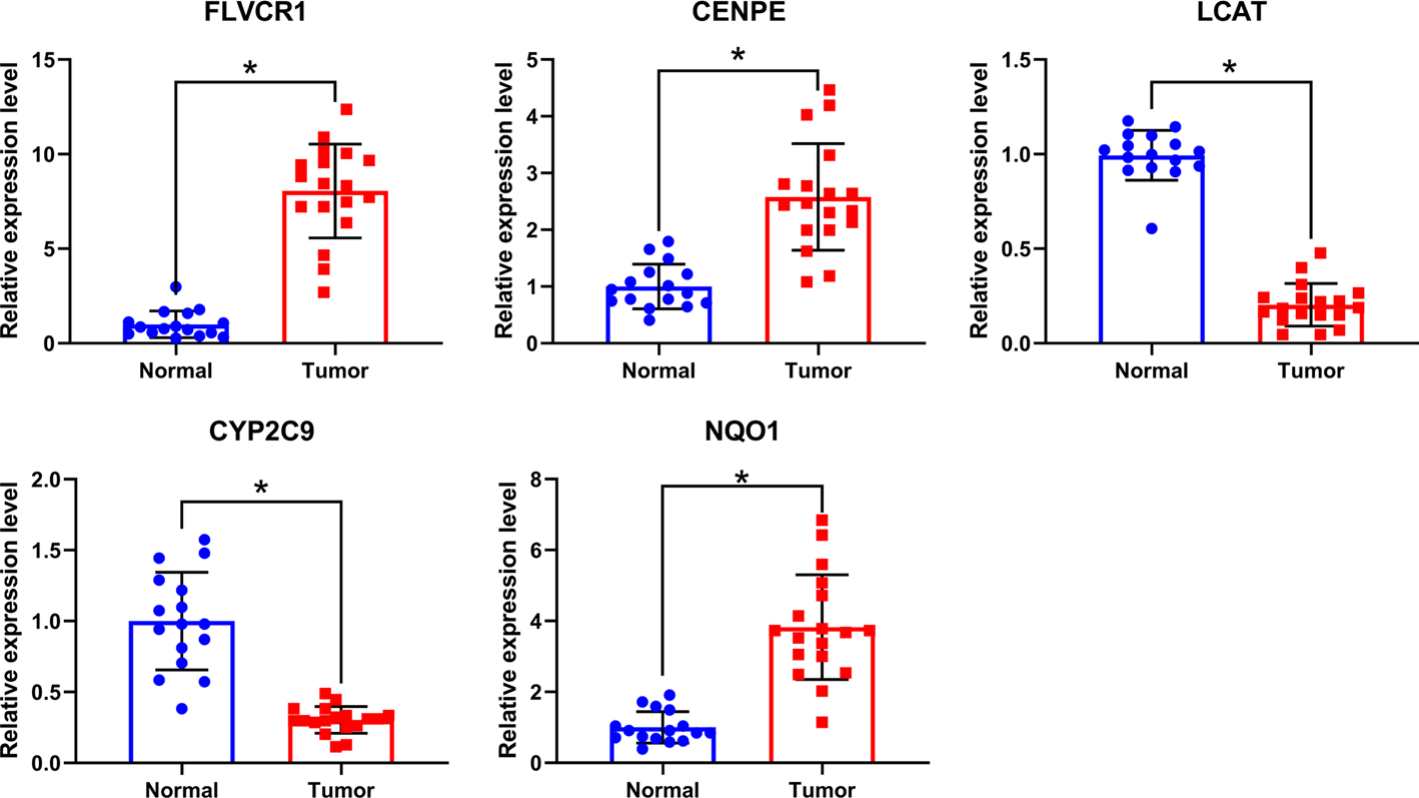
Relative mRNA expression of key CRGs in HCC and normal tissues detected by qPCR. *P < 0.05

Meanwhile, we analyzed the expression of key CRGs in 371 LIHC tissues and 50 normal tissues in the UALCAN online tool. The results revealed FLVCR1, CENPE and NQO1 were significantly higher expressed in HCC group, while LCAT and CYP2C9 were significantly lower compared with the control group (Fig. 3). To confirm the expression of key CRGs, we collected HCC and normal tissues from Lanzhou University Second Hospital, and performed qPCR to detect the relative mRNA expression of key CRGs. The results were consistent with online data. The results suggested that FLVCR1, CENPE and NQO1 had a higher expression, while LCAT and CYP2C9 had a lower expression compared with the normal group (Fig. 4).

### Development and validation of the CRRS prognostic model

**Fig. 5:**
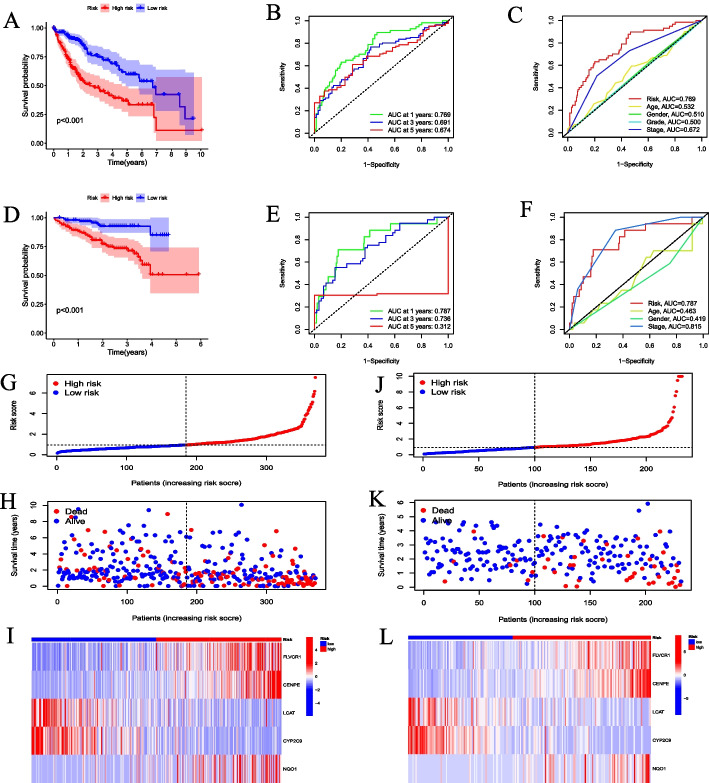
Evaluation and validation of the survival and risk of CRRS model. **A**, **D** The survival analysis of CRRS model in the TCGA-LIHC and ICGC-LIRI-JP datasets. **B**, **E** The ROC analysis of CRRS model in the TCGA-LIHC and ICGC-LIRI-JP datasets. **C**, **F** The ROC curve analysis of the CRRS model and clinical indicators in the TCGA-LIHC and ICGC-LIRI-JP datasets. **G-I** The distribution of risk score, survival status, and the heatmap of the 5 genes in the TCGA-LIHC dataset. **J-L** The distribution of risk score, survival status, and the heatmap of the 5 genes in the ICGC-LIRI-JP dataset

To evaluate and validate the predictive potential of the risk score prognostic model, we analyzed the differences in survival between the high-risk and low-risk groups. The results showed that the OS of the high-risk group was shorter than that of the low-risk group (*P* < 0.001) in the TCGA and ICGC datasets (Fig. 5A and D). The time-dependent ROC curve demonstrated that the AUC values for 1 -, 3 -, and 5-year OS in the TCGA dataset were 0.769, 0.691, and 0.674, respectively (Fig. 5B). Similarly, in the ICGC dataset, the AUC values for 1-, 3-, and 5-year OS were 0.787, 0.736, and 0.312, respectively (Fig. 5E). Furthermore, in the ROC curve containing risk score, age, gender, stage, and grade, the AUC value of the risk score was higher than that of other indicators in the TCGA dataset (Fig. 5C). In the ICGC dataset, the AUC value of the risk score was higher than that of other indicators except for stage (Fig. 5F). In addition, as the risk score increased, so did the number of deaths. Compared with the low-risk group, FLVCR1, CENPE, and NQO1 were highly expressed in the high-risk group, while LCAT and CYP2C9 were lowly expressed (Fig. 5G–I). The results of ICGC were consistent with TCGA (Fig. 5J–L).

### Correlation of prognostic model risk score with clinical indicators

**Fig. 6:**
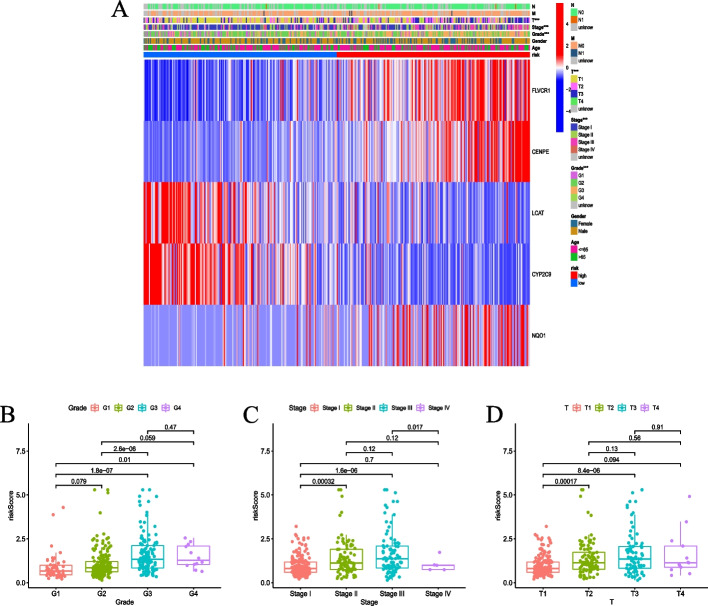
The risk score with the clinical indicators in the TCGA-LIHC dataset. **A** The heatmap for the 5 genes based on risk score and clinical indicators, ***P < 0.001. **B** The boxplot of risk score based on CRGs in HCC patients with different grades. **C** The boxplot of risk score in HCC patients with different tumor stages. **D** The boxplot of risk score in HCC patients with different T stages

To determine whether the risk score prognostic model is applicable to HCC patients with different clinical characteristics, we analyzed the correlation between risk score and age, gender, pathological grades, tumor stages, and TNM stages, and identified a significant correlation between risk score and pathological grades, tumor stages, and T stages in the TCGA-LIHC dataset (*P* < 0.001) (Fig. 6A). The Kruskal–Wallis test showed that there were differences in risk score among pathologic grades, tumor stages, and T stages, although the differences in the risk score among some subgroups were not statistically significant (*P* > 0.05). The risk score tended to rise with pathological grades, tumor stages, and T stages (Fig. 6B–D).

### Establishment and assessment of the nomogram for survival prediction

**Fig. 7:**
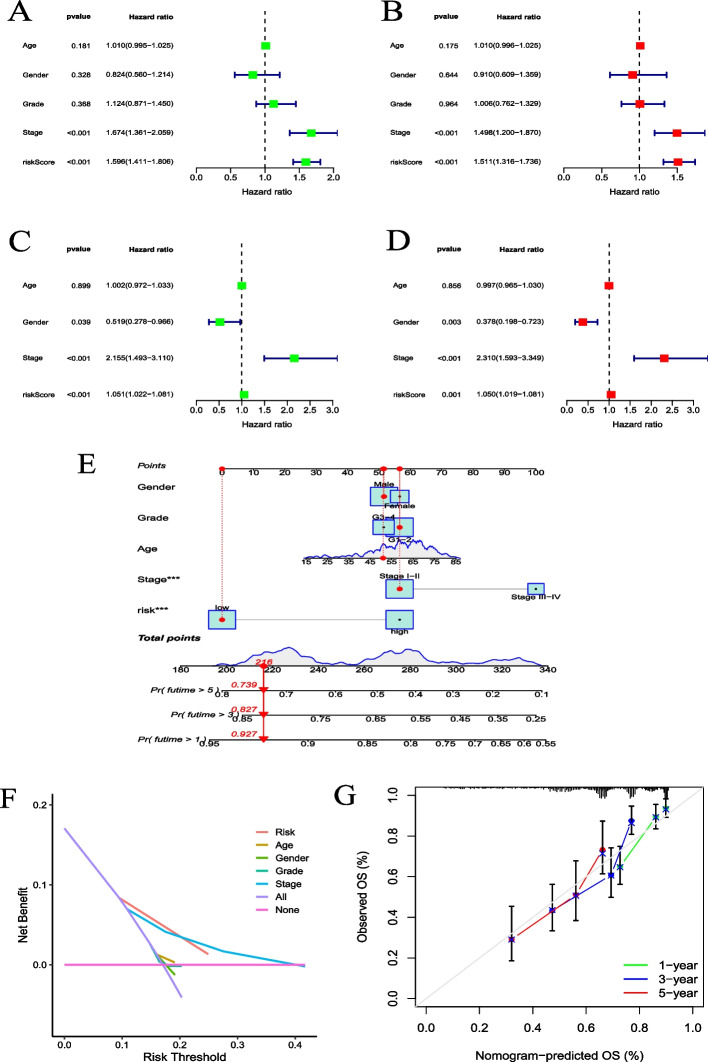
Construction and validation of the clinical prognostic model. **A**, **B** Univariate and multivariate Cox regression analyses based on risk score and other clinical indicators in the TCGA-LIHC dataset. **C**, **D** Univariate and multivariate Cox regression analyses based on risk score and other clinical indicators in the ICGC-LIRI-JP dataset. **E** The nomogram for predicting the probability of 1-, 3-, and 5-year OS for HCC patients. **F** The DCA of the 1-year survival probability in the TCGA-LIHC dataset. **G** The calibration curve of the nomogram for predicting 1-, 3-, and 5-year survival probability. ***P < 0.001

Since CRRS was significantly associated with the malignancy of HCC, we determined whether CRRS was a clinically independent prognostic factor for HCC patients by univariate and multivariate Cox regression analyses. As predicted, the results suggested that CRRS is an independent prognostic factor for HCC (Fig. 7A–D). Subsequently, based on the TCGA dataset, we further constructed the nomogram of risk score, age, gender, grade, and stage, which provided a visual method for predicting the 1 -, 3 -, and 5-year survival probability of HCC patients (Fig. 7E). The DCA showed that CRRS has a higher clinical net benefit than other clinical indicators (Fig. 7F). The calibration curve displayed that there was a good agreement between the survival probability predicted by the nomogram and the actual observed probability (Fig. 7G). These results suggested that the established nomogram has a good prognostic value for HCC patients.

### Function and pathway enrichment analysis based on the CRRS model

**Fig. 8:**
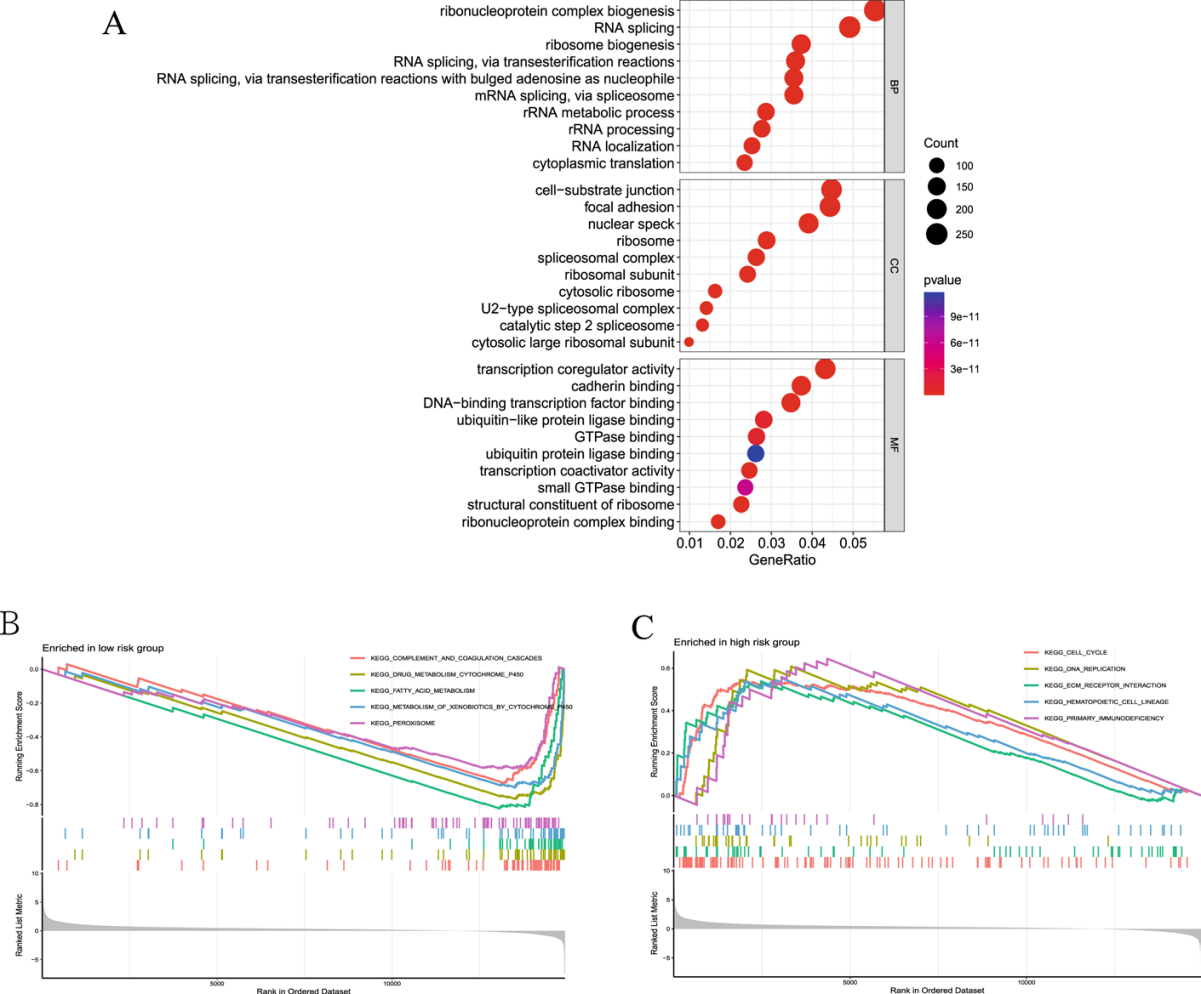
Function enrichment analysis. **A** GO enrichment analysis. The size of the circle indicates the number of genes. The screening criterion was set as adjusted *P* < 0.05. **B-C** Enrichment plots from GSEA analysis in the low-risk and high-risk groups. For more information about KEGG pathway, see https://www.kegg.jp/kegg/kegg1.html

To further explore the differences in the gene functions and pathways between the subgroups classified by CRG risk score, we identified the 5707 DEGs between the high-risk and low-risk groups, which mainly enriched in the ribosome and spliceosome. Biological process involved ribosome biogenesis, RNA splicing, and cytoplasmic translation. The cellular component mainly focused on ribosome and spliceosome. The molecular function analysis showed that most of genes were involved in transcription coregulator activity, cadherin binding, ubiquitin-like protein ligase binding, GTPase binding, and ribonucleoprotein complex binding (Fig. 8A).

GSEA analysis indicated that the most enriched pathways in the low-risk group were complement and coagulation cascades, drug metabolism cytochrome p450, fatty acid metabolism, metabolism of xenobiotics by cytochrome p450, and peroxisome (Fig. 8B). In contrast, cell cycle, DNA replication, ECM receptor interaction, hematopoietic cell lineage, and primary immunodeficiency were enriched in the high-risk group (Fig. 8C).

### Immune landscape of CRRS

**Fig. 9:**
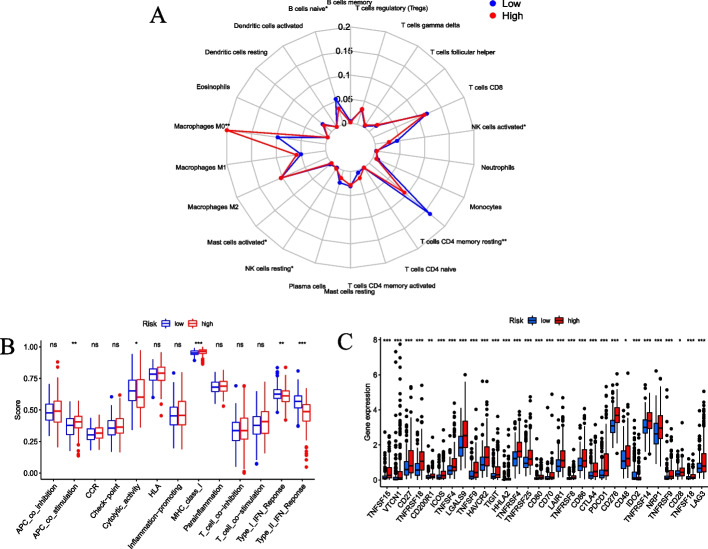
Risk score and TME. **A** The radar map of the 22 immune cells. **B** The boxplot of immune-function score in high-risk and low-risk groups. **C** The differences in immune checkpoint genes between high-risk and low-risk groups. *P < 0.05, **P < 0.01, ***P < 0.001

We investigated the immune cell differences between the high-risk and low-risk groups, and revealed that compared with the low-risk group, Macrophages M0 was significantly higher expressed in the high-risk group, while CD4^+^T cells memory resting, NK cells activated, and B cells naive were significantly lower (Fig. 9A). Accordingly, we analyzed the immune-related functions, and found that cytolytic activity, type I IFN response, and type II IFN response were enriched in the low-risk group, APC co-stimulation and MHC class I response were enriched in the high-risk group (Fig. 9B). Based on the above results, we speculated that this risk score model is involved in immune microenvironment regulation. In addition, we found that the expression levels of immune checkpoint genes in the high-risk group was generally higher than that in the low-risk group (Fig. 9C).

## Discussion

The occurrence of HCC is a complex biological process. It is particularly important to explore the pathogenesis of HCC, find new biomarkers at the molecular level, and achieve early diagnosis and treatment [13]. Abnormal coagulation in cancer patients causes cancer-related thrombosis, which is one of the leading causes of cancer-related death, resulting in human suffering and loss of life. The change in coagulation function plays a vital role in the invasion and metastasis of tumor cells [14]. The research on inhibiting the coagulation system to reduce tumor growth and metastasis has attracted much attention [15]. However, little attention has been paid to the relationship between coagulation and prognosis, immune microenvironment of HCC. Therefore, we aimed to elucidate the role of CRGs in HCC tumor development, prognostic assessment, and immune microenvironment.

In this study, we identified 5 key CRGs to construct a prognostic model. The prognostic model showed that FLVCR1, CENPE and NQO1 were risk factors, while LCAT and CYP2C9 were protective factors. Meanwhile, compared with the control group, FLVCR1, CENPE and NQO1 were significantly higher expressed in HCC, while LCAT and CYP2C9 were significantly lower expressed. According to growing evidence, CRGs are associated with the prognosis of many malignancies. Studies have shown that FLVCR1 plays a crucial role in various biological processes such as cell proliferation and apoptosis, and is significantly highly expressed in HCC, which is associated with increased cell proliferation and invasion [16]. FLVCR1 can promote the proliferation of synovial sarcoma by inhibiting apoptosis and autophagy [17]. NQO1 encodes a reductase that regulates oxidative stress of chromatin binding-proteins for DNA damage in cancer cells [18]. NQO1 is significantly up-regulated in HCC patients and expected to be a therapeutic target and prognostic marker for HCC [19]. CYP2C9 is a member of the cytochrome P2C (CYP2C) family and is involved in clinical drug metabolism. The low expression of CYP2C9 and LCAT in HCC is associated with poor prognosis [20–22]. Nevertheless, the research on the expression of CENPE in HCC samples and its clinical relevance is very limited. One study using human hepatoma cell lines, animal models, and human liver cancer samples found that the low expression of CENPE contributes to the development of HCC [23], which is inconsistent with this study. This may be related to chromosome instability caused by the decrease of CENPE. Whether CENPE has a two-sided effect in the occurrence of HCC needs a large number of experiments to further study.

HCC patients were divided into two groups based on risk score. FLVCR1, CENPE, and NQO1 were highly expressed in the high-risk group, while LCAT and CYP2C9 were highly expressed in the low-risk group. This further suggested that CRGs may be the driver genes of HCC development. The survival curve showed that CRRS was correlated with OS, and the OS in the high-risk group was significantly shorter than that in the low-risk group. The ROC curve showed that the CRRS effectively predicted the OS of HCC patients. Univariate and multivariate Cox regression analyses showed that CRRS was an independent prognostic factor. It is suggested that clinical indicators should be considered when screening prognostic features [24]. To improve the prediction accuracy of the model, we incorporated risk score and clinical indicators to construct a nomogram, which made the prognosis more quantitative and intuitive. A recent study revealed that CRGs can predict the prognosis of patients with cutaneous melanoma [25].

To explore possible intrinsic differences in different prognosis of HCC, we performed enrichment analysis and immune analysis. Functional enrichment involved ribosome and spliceosome. GSEA analysis indicated cell cycle, DNA replication, ECM receptor interaction, hematopoietic cell lineage, and primary immunodeficiency were enriched in the high-risk group. It is well known that dysregulation of these processes will lead to the occurrence and metastasis of tumors [26–30]. The relationship between immune cells and tumors is extremely complex. More and more studies have shown that tumor-infiltrating immune cells can affect the efficacy of HCC immunotherapy [31, 32]. HCC cells reshape the tumor microenvironment through various mechanisms, enabling tumor cells to evade immune surveillance by reducing the number of T cells and NK cells, ultimately promoting tumor proliferation and metastasis [33]. In this study, macrophages M0 was significantly higher expressed in the high-risk group, while CD4^+^T cells memory resting, NK cells activated, and B cells naive were significantly lower. Meantime, immune functions showed that cytolytic activity, type I IFN response, and type II IFN response were enriched in the low-risk group. This all indicated immune tolerance in patients with high-risk HCC. These findings contribute to our further understanding of the pathogenesis of HCC. Studies have proven that high expression of immune checkpoint genes can suppress immune activation and promote tumor immune escape [34]. Immune checkpoint blockers are a promising treatment for a variety of malignancies, which can enhance the anti-tumor immune response by restoring immune recognition and immune attack killing. In this study, we found that the risk score of the CRGs model was positively correlated with the expression levels of almost all immune checkpoint genes, which suggested that the higher the risk score, the more likely it is to benefit from immunotherapy. In a similar study [35], two coagulation pathways were collected from the KEGG database and DEGs were analyzed between the two coagulation pathways in HCC patients. Then, 11 CRGs were identified and a CRRS prognostic model was established in the TCGA dataset. Further, this study suggested that there has correlation between coagulation and TME in HCC, and the risk score can serve as a prognostic biomarker for predicting HCC survival and guiding immunotherapy.

### Limitations

In addition, this study also had some limitations. Firstly, due to the lack of complete clinicopathological information, we collated some clinical data for analysis. Secondly, although the relationship between CRGs and HCC prognosis has been found in HCC patients, the mechanism behind these phenomena remains unclear, and a large number of experiments are still needed to further study the role of CRGs in HCC.

## Conclusions

In this study, we identified 5 key CRGs associated with HCC. The CRRS prognostic model constructed based on CRGs can effectively predict the prognosis of HCC patients. The combination of the risk score with other clinical indicators increased its clinical application potential. The risk score was also correlated with the TME. In addition, these HCC-associated CRGs may become new targets for the diagnosis or treatment of HCC.

## Supplementary Information

**Additional file 1: Table S1.** The clinical information of HCC samples in the TCGA dataset and the ICGC dataset.

**Additional file 2: Table S2.** List of primers.

## Data Availability

The datasets used in this study can be found in the GEO database (https://www.ncbi.nlm.nih.gov/geo/), TCGA database (https://portal.gdc.cancer.gov/) and ICGC database (https://dcc.icgc.org/). The CRGs were obtained from GeneCards database (https://www.genecards.org/). Further inquiries can be directed to the corresponding author.
