# Supplementary material for: Development and validation of a coagulation-related genes prognostic model for hepatocellular carcinoma
Source: BMC Bioinformatics. 2023 Mar 9;24:89. doi: 10.1186/s12859-023-05220-4 (PMC9996845; doi:10.1186/s12859-023-05220-4)
Supplement: Supplementary file 1 — Additional file 1: Table S1. The clinical information of HCC samples in the TCGA dataset and the ICGC dataset. [file 12859_2023_5220_MOESM1_ESM.docx]

Table S1 The clinical information of HCC samples in the TCGA dataset and the ICGC dataset.

| Characteristics | TCGA | | ICGC | |
| --- | --- | --- | --- | --- |
|  | Alive | Dead | Alive | Dead |
|  | (N=244) | (N=126) | (N=189) | (N=43) |
| Gender |  |  |  |  |
| Male | 171 (70.1%) | 78 (61.9%) | 144 (76.2%) | 27 (62.8%) |
| Female | 73 (29.9%) | 48 (38.1%) | 45 (23.8%) | 16 (37.2%) |
| Age_group |  |  |  |  |
| Age <65 | 154 (63.1%) | 67 (53.2%) | 68 (36.0%) | 15 (34.9%) |
| Age ≥ 65 | 90 (36.9%) | 59 (46.8%) | 121 (64.0%) | 28 (65.1%) |
| Grade |  |  |  |  |
| G1 | 37 (15.2%) | 18 (14.3%) |  |  |
| G2 | 119 (48.8%) | 58 (46%) |  |  |
| G3 | 80 (32.8%) | 41 (32.5%) |  |  |
| G4 | 7 (2.9%) | 5 (4.0%) |  |  |
| Unknown | 1 (0.4%) | 4 (3.2%) |  |  |
| Stage |  |  |  |  |
| I | 130 (53.3%) | 41 (32.5%) | 35 (18.5%) | 1 (2.3%) |
| II | 60 (24.6%) | 25 (19.8%) | 88 (46.6%) | 18 (41.9%) |
| III | 41 (16.8%) | 44 (34.9%) | 56 (29.6%) | 15 (34.9%) |
| IV | 2 (0.8%) | 3 (2.4%) | 10 (5.3%) | 9 (20.9%) |
| unknown | 11 (4.5%) | 13 (10.3%) |  |  |
| T |  |  |  |  |
| T1 | 136 (55.7%) | 45 (35.7%) |  |  |
| T2 | 65 (26.6%) | 28 (22.2%) |  |  |
| T3 | 38 (15.6%) | 42 (33.3%) |  |  |
| T4 | 3 (1.2%) | 10 (7.9%) |  |  |
| unknown/TX | 2 (0.8%) | 1 (0.8%) |  |  |
| M |  |  |  |  |
| M0 | 183 (75.0%) | 83 (65.9%) |  |  |
| M1 | 1 (0.4%) | 3 (2.4%) |  |  |
| MX | 60 (24.6%) | 40 (31.7%) |  |  |
| N |  |  |  |  |
| N0 | 169 (69.3%) | 83 (65.9%) |  |  |
| N1 | 2 (0.8%) | 2 (1.6%) |  |  |
| NX | 73 (29.9%) | 40 (31.7%) |  |  |
| unknown |  | 1 (0.8%) |  |  |
| Risk group |  |  |  |  |
| Low-risk | 139 (57.0%) | 46 (36.5%) | 93 (49.2%) | 7 (16.3%) |
| High-risk | 105 (43.0%) | 80 (63.5%) | 96 (50.8%) | 36 (83.7) |
