# Supplementary material for: Development and validation of a coagulation-related genes prognostic model for hepatocellular carcinoma
Source: BMC Bioinformatics. 2023 Mar 9;24:89. doi: 10.1186/s12859-023-05220-4 (PMC9996845; doi:10.1186/s12859-023-05220-4)
Supplement: Supplementary file 2 — Additional file 2: Table S2. List of primers. [file 12859_2023_5220_MOESM2_ESM.docx]

Table S2 List of primers.

| Primer | Sequence |
| --- | --- |
| FLVCR1 | Forward: 5′-AGACAGTCCCCCTGAAGAGT-3′ |
|  | Reverse: 5′-GACTGAATAAAAGGCACCAGTCA-3′ |
| CENPE | Forward: 5′-GACCGACAGAACCACCAAGT-3′ |
|  | Reverse: 5′-AGAGCACTTTTCTCTCAGGCT-3′ |
| LCAT | Forward: 5′-TGGATGTGCTACCGCAAGAC-3′ |
|  | Reverse: 5′-CGATCCAGCAGTCTACCCCA-3′ |
| CYP2C9 | Forward: 5′-GGGGCATTATCCATCTTTCACT-3′ |
|  | Reverse: 5′-ACTCTCCGTAATGGAGGTCG-3′ |
| NQO1 | Forward: 5′-TTTGCTTACACTTACGCTGCC-3′ |
|  | Reverse: 5′-AGTACATGGAGCCACTGCCA-3′ |
| GAPDH | Forward: 5′-AGTGGCAAAGTGGAGATT-3′ |
|  | Reverse: 5′-GTGGAGTCATACTGGAACA-3′ |
